# Unexplained Syncope and Diagnostic Yield of Tests in Syncope According to the ICD-10 Discharge Diagnosis

**DOI:** 10.4021/jocmr1569w

**Published:** 2013-10-12

**Authors:** Martin Huth Ruwald, Morten Lock Hansen, Morten Lamberts, Michael Vinther, Christian Torp-Pedersen, Jim Hansen, Gunnar Hilmar Gislason

**Affiliations:** a Department of Cardiology, Gentofte Hospital, Denmark; b Department of Cardiology, Herlev Hospital, Denmark

**Keywords:** Syncope, Diagnostic techniques, Cardiovascular, Etiology

## Abstract

**Background:**

The etiology of syncope according to the discharge diagnosis from hospital admissions has not been examined before. Therefore the aims of this study were to examine the diagnostic yield of tests and frequency of unexplained cases during admission and after workup after an ICD-10 diagnosis of syncope.

**Methods:**

A retrospective chart review of 600 patients discharged with the primary ICD-10 discharge diagnosis of syncope R55.9 was performed. Causes and clinical characteristics of syncope according to the physician were noted both after initial discharge and after workup.

**Results:**

During a mean follow-up period of 2.5 years (SD: ± 1.30) several diagnostic tests were used (mean number of tests per patient was 4.7 (SD: ± -2.0)) and the mean length of admission was 2.1 days (± 1.5).The final diagnosis after workup was reflex syncope in 21%, cardiac 18%, orthostatic hypotension 10%, other causes 4% and unknown/unexplained syncope in 48% with wide age differences. The diagnostic yield of tests was generally low and differed widely depending on usage during admission or usage during subsequent workup.

**Conclusions:**

The underlying etiology of syncope remains difficult to establish despite the high use of diagnostic tests and the diagnostic yield of many tests implemented in the care path is generally low.

## Introduction

Syncope is a common condition associated with frequent hospitalizations [1-4] that is difficult to evaluate and is associated with a high mortality rate in selected subgroups of patients [5-10]. Existing patterns of care are characterized by high diversity, high cost and low diagnostic and therapeutic yield. Between 39% and 50% of admitted patients are discharged without an explanation for syncope [11], and in one study 60% of older patients received no specific therapies during their admission [8]. Etiologic explanation of syncope is important to implement correct therapeutic strategies and for risk stratification. In most patients with syncope, the initial evaluation does not provide a definite diagnosis and additional diagnostic testing is often needed. The strategy for testing is usually clearer when syncope occurs in the presence of severe structural heart disease. However, in the elderly and in patients with suspected, but undiagnosed arrhythmias or vasovagal syncope, elucidation is required for the role of long term ECG recording, implantable loop recorders, tilt-table tests and carotid-sinus massage. The challenge is to reduce the number of patients where syncope remains unexplained. The primary objective of this retrospective study was to explore which etiologic category syncope could be assigned to after discharge and after workup and to estimate the use of diagnostic tests and the diagnostic yield. We hypothesized that, in a contemporary clinical setting, the etiology of syncope after discharge would be difficult to establish even with the use of a wide range of tests.

## Materials and Methods

### Study population

We retrospectively identified a cohort of patients who weredischarged for syncope according to the discharge diagnosis R55.9 (‘syncope and collapse’) from January 1st, 2007 to December 31, 2010 on three University hospitals in the Capital Region of Copenhagen.

A total of 1,223 charts of discharged patient for syncope were identified through the electronic patient management system, 23 charts were insufficient for documentation or the chart could not be accessed by the reviewers. From this overall syncope population of 1,200, we randomly selected 50% (due to resources) from each hospital of the total discharged patients for individual chart review. Of these 600 patients, 570 (95%) satisfied the European Society of Cardiology (ESC) definition of total loss of consciousness and syncope [12] and entered the analysis and baseline model. We have previously validated the R55.9 discharge diagnosis code in the same cohort and in 5,262 other medical patients and found a positive predictive value of 95% and a sensitivity of 63% [13].

All charts from discharged patients were read independently, reviewing all notes from the physician history and physical examination, physician notes during rounds and at discharge, imaging reports (if available), laboratory tests, electrocardiogram (ECG) or echocardiography reports, and any other pertinent investigations (for example, tilt-testing, electrophysiological studies, orthostatic hypotension test or carotid sinus massage). The most likely cause of the syncope according to the discharging physician was noted both after initial admission and after workup.

The workup of each individual patient was reviewed during December 2011 by examining the patient charts from the date of the initial syncope, which had the discharge diagnosis of R55.9 in the study period. The etiology was determined and noted if definite diagnosis was made within the study period otherwise the syncope had to be classified as unknown.

### Hospitals chosen for chart review

Of the three hospitals, one is a major center of cardiology with a specialized syncope unit and catheter laboratory for pacemaker implantations, coronary angiography and cardiac ablations, while the other two are representative of large volume hospitals with large open-referral emergency departments and designated departments of internal medicine and neurology.

### Statistics

Patient demographics (age, sex, pharmacotherapy and comorbidity) were analyzed for all patients identified as having syncope based on the administrative coding in the database. Age, distributed in a non-Gaussian fashion, was expressed as median and interquartile ranges. T-test and non-parametric Mann-Whitney U test were used where appropriate on variables. Comparison between proportions and groups was by means of the chi square test. For patients included in the chart review, information collected on the abstraction form regarding diagnostic and treatment procedures was reported descriptively.

All data management and analyses were conducted using SAS Version 9.2 (SAS Institute, Inc., Cary, NC), and findings with two-sided P-values < 0.05 were considered statistically significant.

### Ethics

The study was approved by the Danish Data Protection Agency (ref. 2007-58-0015, int. ref: GEH-2010-001).

## Results

A total of 600 charts from patients discharged after syncope (ICD-10 diagnosis. R559) were reviewed systematically of which 570 (95%) satisfied the ESC criteria for syncope [12]. The median age was 68.5 years (IQR 53 - 81), and 48% were female. The mean follow-up period was 2.5 years (SD ± 1.30) and the mean length of admission of 2.1 days (± 1.5). A history of hypertension was present in 51%, ischemic heart disease in 22%, atrial fibrillation in 14%, congestive heart failure in 11% and diabetes in 10%. No major differences between the three hospitals were seen in terms of comorbidities or pharmacotherapy and the patient baseline characteristics of comorbidities and pharmacotherapy are presented in Table 1. Of the 570 patients, 249 (44%) presented with prodromal symptoms, primarily represented by “light-headedness”, nausea and sweating accounting for 61%, whereas palpitations, chest pain and exertion accounted for 6%, 4% and 6%, respectively.

**Table 1:** Baseline Characteristics

Characteristics	Total (%)
Number of patients	570 (100)
Men	297 (52)
Age years (IQR)	68.5 (53 - 81)
Previous syncope	130 (23)
Prodromal symptoms	249 (44)
Comorbidities	
Ischemic heart disease	126 (22)
Peripheral vascular disease	78 (14)
Previous myocardial infarction	87 (15)
Systemic hypertension	288 (51)
Previous or current atrial fibrillation	77 (14)
Other arrhythmias	37 (6)
Previous stroke	72 (13)
Congestive heart failure	61 (11)
Previous or ongoing cancer	49 (9)
Chronic obstructive pulmonary disorder	46 (8)
Diabetes	56 (10)
Cardiac pacemaker or ICD unit	21 (4)
Epilepsy	7 (1)
Alcoholism	18 (3)
Dementia	29 (5)
Depression	25 (4)
Previous PCI or CABG	61 (11)
Previous or current smoker	223 (39)
Alcohol intake above recommended level	124 (22)
Pharmacotherapy	
Beta blockers	140 (25)
ACEi/ARB	210 (37)
Digoxin	21 (4)
Nitrates	34 (6)
Calcium channel blockers	85 (15)
Spironolactone	21 (4)
Thiazide	111 (19)
Loop diuretics	85 (15)
Class Ic antiarrhythmic drugs	1 (0)
Class III antiarrhythmic drugs	13 (2)
Anxiolytics	56 (10)
Antipsychotics	27 (5)
Antidepressants	76 (13)
Glucose lowering drugs	51 (9)

Age is given in median and interquartile range (IQR). ACEi: angiotensin converting enzyme inhibitor; ARB: angiotensin receptor blocker; PCI: percutaneous coronary intervention; CABG: coronary artery bypass graft.

### Diagnostic tests

The diagnostic tests used during admission and workup consisted of a wide variety of diagnostic tests, mostly used was ECG (100%), laboratory tests (100%), telemetry (76%), echocardiogram (48%) and long-term ECG monitoring (35%) as listed in Table 2. The diagnostic overall yield of tilt-testing was found to be 47%, 20% using the orthostatic hypotension test and 18% using implantable cardiac monitoring while resting ECG and echocardiogram yielded a diagnosis in 4% of the patients as shown in Table 2. The mean number of tests per patient was 4.7 (SD: ± 2.0).

**Table 2:** The Total Number of Tests Used During First Admission and Workup

Test	Number (%)	Abnormal test (%)	Definite diagnosis Diagnostic yield of test (%)
Total tests	2601	632 (24)	165 (6)
Laboratory and blood results	570 (100)	NA	NA
ECG	569 (100)	235 (41)	22 (4)
Telemetry	431 (76)	103 (24)	18 (4)
Echocardiogram	272 (48)	97 (36)	11 (4)
Long-term ECG monitoring	198 (35)	70 (35)	29 (15)
Stress ECG	43 (8)	6 (14)	2 (5)
Orthostatic hypotension test	114 (20)	31 (27)	23 (20)
Electrophysiological study	29 (5)	3 (10)	3 (10)
Tilt table	60 (11)	34 (57)	28 (47)
Carotid sinus massage	61 (11)	5 (8)	5 (8)
Implantable loop recorder	38 (7)	7 (18)	7 (18)
MRI cerebrum	25 (4)	6 (24)	3 (12)*
CT cerebrum	143 (25)	25 (17)	4 (3)*
Electroencephalogram	48 (8)	10 (21)	10 (21)*

ECG: electrocardiogram; MRI: magnetic resonance imaging; EP: electrophysiological; CT: computed tomography. *These 17 patients were all diagnosed with neurological disease during the workup, primarily epilepsy, thus the diagnostic yield was as counted, but the patients were probably ultimately classified wrongly as syncopal origin.

In 207 patients (35%) the etiological diagnosis was made during the admission. In these patients the diagnostic yield of the echocardiogram, ECG, orthostatic hypotension test and telemetry were markedly higher compared to the entire cohort (Table 3). In 102 patients (18%) an etiological diagnosis was made during workup (initially classified as unknown). In these patients the diagnostic yield of implantable loop recorder, tilt table test and long-term ECG monitoring were markedly higher than in the total cohort (Table 4). Eleven patients who initially received a definite etiological diagnosis by the discharging physician but referred for further workup were subsequently reclassified as unknown after further workup. Comparing Table 3 and Table 4, an etiological diagnosis is much more likely to be made based on an abnormal test performed during workup than an abnormal test performed during the initial admission.

**Table 3:** Number of Tests Performed During First Admission in the 207 (35%) Patients Where the Etiological Diagnosis was Made During Admission

Test	Number (%)	Abnormal test (%)	Definite diagnosis. Diagnostic yield of test during admission (%)
Laboratory and blood results	207 (100)	NA	NA
ECG	207 (100)	83 (40)	17 (8)
Telemetry	144 (70)	44 (31)	16 (11)
Echocardiogram	72 (35)	33 (46)	7 (10)
Stress ECG	7 (3)	1 (14)	1 (14)
Orthostatic hypotension test	32 (16)	20 (63)	15 (47)
Electrophysiological study	5 (2)	1 (20)	1 (20)
Carotid sinus massage	10 (5)	2 (20)	2 (20)
CT cerebrum	29 (14)	9 (31)	3 (10)*

*These 3 patients received another definitive diagnosis than syncope and were ultimately classified wrongly by the physician.

**Table 4:** Number of Tests Performed During Workup in 102 Patients Where the Etiological Diagnosis Was Unknown at Discharge but Apparent After Workup

Test	Number (%)	Abnormal test (%)	Definite diagnosis. Diagnostic yield of test (%)
Echocardiogram	74 (73)	25 (34)	5 (7)
Long-term ECG monitoring	72 (71)	32 (44)	19 (26)
Stress ECG	13 (13)	1 (8)	1 (8)
Orthostatic hypotension test	30 (29)	8 (27)	7 (23)
Electrophysiological study	10 (10)	2 (20)	2 (20)
Tilt table	36 (35)	25 (69)	22 (61)
Carotid sinus massage	36 (35)	3 (8)	3 (8)
Implantable loop recorder	12 (12)	6 (50)	6 (50)
MRI cerebrum	10 (10)	4 (40)	3 (75)*
CT cerebrum	26 (25)	5 (19)	4 (15)*
Electroencephalogram	13 (13)	4 (31)	4 (31)*

*These 11 tests were performed in patients who received another definitive diagnosis than syncope and were initially classified wrongly by the discharging physician.

### Etiology

During the course of workup 24 (4%) were ultimately diagnosed with conditions mimicking syncope, primarily epilepsy (10 patients).

Table 5 shows the various etiologies of syncope after admission and after workup, respectively. Final diagnosis after workup was reflex syncope 21%, cardiac cause 18% represented by severe brad arrhythmias or blocks 12%, ventricular arrhythmias 3% and other cardiac causes 3%. Orthostatic hypotension accounted for 10%, other non-syncopal causes 4% and ultimately 48% remained of unknown etiology. All accounted etiologies were significantly different comparing before and after workup. Table 5 furthermore shows the age-dependent variation in etiology. When dichotomized at the age of 65 years, reflex syncope is significantly more prevalent in the younger, orthostatic and cardiac causes in the elderly while the difference in prevalence of other causes and unknown etiology remain insignificant.

**Table 5:** Etiology of Syncope After Discharge and After Workup

Etiology	Number (%) after discharge	Number (%) after workup	P value	Age < 65 years	Age ≥ 65 years	P-value between age groups
Reflex^1^	87 (15)	117 (21)	< 0.001	80 (68)	37 (32)	< 0.001
Orthostatic hypotension^2^	41 (7)	55 (10)	< 0.001	15 (27)	40 (73)	0.022
Cardiac	72 (11)	102 (18)	< 0.001	19 (19)	83 (81)	< 0.001
Ventricular arrhythmias	15 (3)	18 (3)				
Sick sinus syndrome	14 (2)	20 (4)				
Advanced second- or third-degree AV block	27 (5)	44 (8)				
Structural cardiac disease	8 (1)	12 (2)				
Other cardiogenic^3^	8 (1)	8 (1)				
Unknown	363 (64)	272 (48)	< 0.001	115 (42)	157 (58)	0.808
Non syncopal events^4^	7 (1)	24 (4)	< 0.001	9 (38)	15 (62)	0.666

^1^Vasovagal, situational and carotid sinus syncope; ^2^Primary autonomic failure, secondary autonomic failure, drug induced and volume depletion;^3^Pulmonary embolism, angina and left main stenosis, digoxin intoxication and pacemaker induced tachycardia;^4^Epilepsy, psychogenic pseudosyncope, stroke, intracerebral tumor, fatigue and collapse.

At the end of diagnostic evaluation, a diagnosis of primary cardiac etiology was established in 15%, 26% and 15% at the three hospitals respectively (including referrals to other hospitals-data not shown).

Table 6 shows the proportions of selected comorbidities and pharmacotherapy according to the result of the diagnostic workup. High amount of cardiovascular comorbidity was noted in the group of cardiac etiology and very low rate of cardiovascular comorbidity was seen in the group of reflex syncope. Comparable rates of cardiovascular medication and comorbidity is noted in the group of unknown diagnosis and cardiac diagnosis.

**Table 6:** Selected Pharmacotherapy and Comorbidity According to Diagnosis After Workup

Pharmacotherapy and Comorbidity	Unknown N = 272	Cardiac N = 102	Orthostatic N = 55	Reflex N = 117	Other causes N = 24
Comorbidity (in %)					
CHF	28 (10)	24 (23)	6 (11)	2 (2)	1 (4)
IHD	67 (24)	39 (38)	11 (20)	6 (5)	5 (20)
AMI	47 (17)	27 (28)	9 (16)	4 (3)	0 (0)
AF	39 (14)	25 (24)	5 (9)	7 (6)	2 (8)
Other arrhythmias	12 (4)	15 (14)	4 (7)	4 (3)	2 (8)
Stroke	34 (12)	19 (18)	12 (22)	3 (2)	4 (15)
Diabetes	21 (8)	15 (15)	12 (22)	5 (4)	2 (8)
Cardiac device	10 (4)	6 (6)	3 (5)	2 (2)	0 (0)
Depression	12 (4)	6 (6)	6 (11)	1 (1)	0 (0)
Pharmacotherapy (in %)					
Beta blockers	67 (25)	41 (39)	18 (33)	14 (12)	1 (4)
ACE/ARB	104 (38)	53 (51)	27 (49)	20 (17)	8 (31)
Thiazide	54 (20)	18 (17)	14 (25)	18 (15)	7 (27)
Loop diuretics	40 (15)	30 (29)	11 (20)	3 (3)	2 (8)
Anxiolytics	33 (12)	9 (9)	8 (15)	3 (3)	3 (12)
Antipsycotics	14 (5)	3 (3)	6 (11)	2 (2)	2 (8)
Antidepressants	43 (16)	-12	11 (20)	8 (7)	3 (12)

CHF: congestive heart failure; IHD: ischemic heart disease; AMI: acute myocardial infarction; AF: atrial fibrillation; ACE: angiotensin converting enzyme; ARB: angiotensin receptor blocker.

Light-headedness, nausea or sweating as a predominant symptom was significantly (P < 0.001) associated with a final etiological diagnosis of reflex syncope, while no significant association was found between cardiac etiology and chest pain or palpitations, probably due to low absolute numbers (Table 7).

**Table 7:** Predominant Prodromal Symptom and Final Etiological Diagnosis

Predominant prodrome	Etiology				
	Cardiac	Orthostatic	Reflex	Unknown	P value
Palpitations (%)	2 (2)	0	2 (2)	12 (4)	0.045
Light-headedness, nausea and sweating (%)	19 (19)	16 (29)	58 (50)	54 (20)	< 0.001
Chest pain (%)	1 (1)	3 (5)	0	5 (2)	0.330
During exercise (%)	3 (3)	0	0	9(3)	0.527

## Discussion

Major findings of this study were the relatively low diagnostic yield and the high number of unexplained cases. This supports previous studies in unselected cohorts, but we approached patients with syncope according to the diagnostic coding used in most countries, which is a major difference and this study should be interpreted with this fact in mind. The general evaluation of a patient with syncope involves a myriad of diagnostic tests, but the annual cost of syncope related admissions is very hard to calculate as no administrative coding is specifically designed for all syncope. It is, however, estimated that the annual costs of syncope related admissions in the US exceeds $2 billion [14]. Some clinical risk stratification algorithms have been developed to identify the high-risk patients but none are widely implemented [15-17]. On the other hand, much evidence points to the very limited utility of performing some tests, such as the basic laboratory tests, cerebral computed tomography, cerebral magnetic resonance imaging and electroencephalography, which, in these unselected patients, have a diagnostic yield of 1% or even less, if used as primary diagnostic tools [18]. The routine use is discouraged by scientific guidelines [12]. Despite this, we find in our study that they are still frequently performed, suggesting that current practice is not in concordance with current guidelines. Concordantly we found a relatively high amount of diagnostic test being used during workup of these patients. We found resting ECG and long-term ECG monitoring to have relatively low diagnostic yield as comparable to previous studies [18-20]. Important variations in diagnostic yield were evident comparing those who received definite diagnosis during admission and those who received definite diagnosis during the workup (Tables 3. 4). This relationship reflects the stepwise selection of patients, but importantly it is noteworthy that in our study the implantable loop recorder and tilt table test proved useful in the workup. The relatively high yield of EEG, MRI and cerebral computed tomography was only due to a definite diagnosis of neurological origin. The diagnostic yield of these tests in terms of a final etiological cause for syncope was 0 for all three tests. Ultimately only 5% received a diagnosis covered by a syncope-mimicking disease, such as epilepsy. We suggest that more syncope-mimicking conditions may reside among the large proportion on unexplained cases as proposed by others [21, 22], which could justify these tests for neurological mimics of syncope in a few selected patients. Interpretation of advanced tests and correct prioritizing of health care resources calls for multidisciplinary syncope-management clinics and close collaboration among medical specialties as suggested by Shen et al [23]. Such units have been advocated in order to access expertise and ensure fast diagnostics and risk stratification at lower costs [12, 24, 25].

The relatively high diagnostic yield of tilt-table test was probably due to a selection process towards healthier patients, where vasovagal syncope was the most likely cause of the syncope and since no clearly defined criteria was used some false positive tilt tests may also have biased the results. Furthermore our relatively low diagnostic yield of implantable loop recorder was probably due to a similar selection of patients, where the etiological diagnosis was the most difficult to establish and the rate of recurrent syncope was very low. Major differences in the diagnostic yield of the tests relied on

### Implications of unknown etiology

The other major finding of the study was that only half of the patients discharged with the diagnosis of syncope received a definite etiological diagnosis despite workup and employment of numerous diagnostic tests. Important in this study is that the patients were selected according to the discharge diagnosis R55.9 leaving out patients who at discharge received other diagnoses. Some of these patients may already have been etiologically diagnosed, biasing these results towards a higher proportion of unknown etiology compared to other studies.

Previous smaller retrospective and prospective studies which did not use the ICD-10 discharge diagnosis have however reached similar results [1, 24, 26, 27]. Chen et al found that multiple potential causes of syncope are common and particularly in the elderly, the exact etiological diagnosis is hard to establish [28]. In our study the percentage of unknown etiology diminishes after workup but only by a decrease of 17% in absolute numbers. This may be caused by our case definition that the discharging physician after discharge or workup had to account for etiology and if not claimed in the chart the case was classified as unknown. We decided not to second-guess the evaluation of the clinicians. Also, importantly as previously stated, the discharge diagnosis R55.9 does not cover all cases of syncope. The R55.9 coding covers around 63% of the cases, thus a relatively large proportion receive other relevant diagnoses at discharge, which may bias our results towards even lower diagnostic yields. Furthermore we found no obvious age-dependent variation suggesting that the younger patients are easier to diagnose, but along with studies previously cited we found increasing prevalence of orthostatic and cardiac syncope in the elderly and more reflex syncope in the young.

It is therefore noteworthy that in the present study comparable rates of cardiovascular medication and comorbidities are seen in the group of undiagnosed patients (unexplained etiology) and in the group of cardiac etiologies. An extrapolation of these findings may indicate that a large proportion of patients in our study with unexplained syncope may have “covert” cardiac etiology and are at an increased risk of arrhythmogenic death. Further research is needed to clarify the implications of this finding, but it emphasizes the need to establish an etiological diagnosis.

### Limitations

First, this is a retrospective study of hospitalized inpatient databases and must be weighted as such. A majority of syncope cases are treated in outpatient and general practice settings and generalizing our findings to outpatient or general practice databases should not be done. Therefore, data from those individuals who do not seek medical attention or who are only seen in outpatient clinic or offices are not captured. For syncope, this results in a slight bias to more severe cases of syncope because patients with milder/less severe symptoms may not seek medical attention.

Second our ‘‘gold standard’’ relied solely on chart documentation. However, when discharged from the hospital, patients tend to have more extensive workup. Therefore, the charts most likely reflected the ‘‘true diagnosis’’ but due to the retrospective observational character of this study no pre-specified criteria was used for correct diagnosis of a given test and was left to the current practice of the department. Third, our case definition of syncope only included 63% of all patients with syncope according to our validation study, which essentially may bias the results towards higher proportions of unexplained syncope compared to other settings and syncope populations. Fourth, patients who at discharge could be classified as cardiac syncope and orthostatic hypotension syncope should not even have received the R55.9 discharge diagnosis but should have been given another concrete ICD-10 diagnosis (namely, I95.1, I45.x and I49.x, and so on) accompanied by R55.9 as a secondary diagnosis. Finally, we could not account for factors that might have influenced the attending physician in ordering certain tests or establishing the etiology of syncope.

### Conclusion

In conclusion we found that the discharge diagnosis of R55.9 covers a wide variety of etiological manifestations, but mostly, it covers syncope due to unknown cause even after workup. The diagnostic yield of many tests implemented in the care path is generally low and more studies are needed for a better selection of patients.

## Acknowledgement

Grants and funding sources, The Danish Heart Association, The Lundbeck Foundation, Snedkermester Sophus Jakobsens Fond, Arvid Nilssons Fond, Helsefonden and Knud Hoejgaards Fond.
